# Effect of Alternative Splicing Euchromatic Histone Lysine Methyltransferase 2 (*EHMT2/G9A*) on Spermatogenesis in Mongolian Horses

**DOI:** 10.3390/ani15081106

**Published:** 2025-04-11

**Authors:** Tergel Baatar, Dailing Song, Yajuan Weng, Guoqing Wang, Liangyi Jin, Rui Guo, Bei Li, Manglai Dugarjaviin

**Affiliations:** 1 Key Laboratory of Equus Germplasm Innovation (Coconstruction by Ministry and Province), Ministry of Agriculture and Rural Affairs, Hohhot 010018, China; sarenterigele@126.com (T.B.); sdailing@163.com (D.S.); wengyajuan1@163.com (Y.W.); gq101220jy@163.com (G.W.); 15575532582@163.com (L.J.); gr120225yjs@163.com (R.G.); 2 Inner Mongolia Key Laboratory of Equine Science Research and Technology Innovation, Inner Mongolia Agricultural University, Hohhot 010018, China; 3 Equus Research Center, College of Animal Science, Inner Mongolia Agricultural University, Hohhot 010018, China

**Keywords:** alternative splicing, *EHMT2/G9A*, spermatogenesis, Mongolian horse

## Abstract

The epigenetic regulation of gene expression through the covalent modification of histones is crucial for developing germline cells. To study the regulatory role of alternative splicing (AS) of euchromatic histone lysine methyltransferase 2 (EHMT2/G9A) in spermatogenesis in Mongolian horses, this study first examines the localization of the *EHMT2* gene in testicular support cells and then predicts the higher-order structures of sequences with and without AS. Two types of lentiviral vectors for overexpression were subsequently constructed for the *EHMT2* gene, one with AS and one without, to infect support cells. The proliferation and activity of infected cells were measured using CCK8, and the differential expression of spermatogenesis-related genes in the two types of support cells was analyzed via qRT–PCR. We analyzed the expression of *EHMT2* by immunofluorescence staining. *EHMT2* was expressed in the nuclei of Sertoli cells. The expression of spermatogenesis-related genes was measured in the two types of cells. The results reveal that the expression levels of the *FSH*, *Stra8*, *CCNB2*, *CDC27*, *NRG1*, *PPP2R5C*, *CCNB2*, and *YWHAZ* genes in the AS group were greater than those in the control group. These results indicate that AS events in *EHMT2* affect gene expression and thus affect spermatogenesis.

## Simple Summary

In this study, the localization of the *EHMT2/G9A* gene in Sertoli cells was examined, the higher-level structures of the two splice variants of *EHMT2* were predicted, two lentiviral vectors to infect Sertoli cells were constructed, proliferation and activity were evaluated, and the differential expression of sperm-related genes was measured.

## 1. Introduction

The Mongolian horse is a unique breed; it is one of the oldest breeds of domestic horse, serving as a significant genomic resource [1], with strong adaptability and resistance to harsh climates and challenging feeding conditions [2]. Currently, Mongolian horses are predominantly found in regions of northeastern and northern China, particularly Inner Mongolia and Mongolia, and certain eastern Russian areas. There has been extensive research on the athletic ability [3], genetic evolution [4], and gut microbiota [5] of Mongolian horses, as well as on male fertility [6] and female reproductive capabilities [7] in this breed.

Male fertility is crucial for the survival of the Mongolian horse species. Spermatogenesis is not only one of the most critical biological processes for male fertility, but also a definitive determinant of reproductive success, holding significant implications in the livestock industry. Spermatogenesis is a highly complex and coordinated process. The process is divided into the spermatogonia proliferation phase, spermatocyte meiotic phase, and spermiogenesis phase [8]. Sertoli cells are somatic cells of the testis and are essential for testicular formation and spermatogenesis. Sertoli cells promote the progression of germ cells to sperm by coming in contact with and controlling the environment within the seminiferous tubules [9]. Additionally, Sertoli cells play an essential role in testis development and normal spermatogenesis by providing support and nutrients.

Alternative splicing (AS) is critical for the posttranscriptional regulation of gene expression. Studies have shown that approximately 70% of human genes may undergo AS, and up to half of these alter the reading frame of the transcript [10]. AS notably expands the function and form of the genome of organisms with limited gene numbers and is especially important in highly complex tissues and organisms [11,12]. AS constitutes a crucial posttranscriptional regulatory mechanism for gene expression, enabling the generation of multiple mature messenger RNAs (mRNAs) from a single precursor mRNA through the process of selective splicing [13,14].

AS plays a key role in sperm production in male mammals. The progression of spermatogenesis along specific developmental trajectories depends on the coordinated regulation of AS at the post-transcriptional level. It ensures the normal production of sperm by regulating the expression of genes related to spermatogenesis, testicular development, or the development of germ cells. Given the consisting of diverse cell types and biological processes, spermatogenesis is an excellent model for studying gene regulation at the transcriptional and post-transcriptional levels [15]. For example, ESRP1, a specific RNA-binding protein found in epithelial cells, has been implicated in mouse oogenesis and female fertility through its role in regulating mRNA AS [16], and, as a key alternative splicing regulator, Ptbp2 plays an important role in male fertility and male germ cell survival by regulating the AS of germ cell mRNAs in the testis [17]. Studies have shown that AS of hormone receptor genes affects male infertility. Recently, research has demonstrated that the RNA helicase DDX5 plays crucial post-transcriptional roles in sustaining and facilitating the function of spermatogonia by regulating the splicing of functional genes within these cells [18].

Epigenetic regulation of gene expression by covalent modification of histones is critical for germline cell development. In mammals, histone H3 lysine 9 (*H3K9*)-specific histone methyltransferases (HMTases), such as *G9a/EHMT2*, *SETDB1*, and *SUV39H*, play important roles [19]. It has been reported that male and female germline cells of *G9a*/*EHMT2* mutant mice cannot undergo meiosis, indicating an essential role of *G9a/EHMT2* during reproduction [20]. Importantly, in previous transcriptome sequencing studies, the *EHMT2* gene in Mongolian horses was shown to undergo exon skipping events, and the expression level in the testicular tissue of mature Mongolian horses was significantly greater than that in immature testicular tissue [21]. This phenomenon suggests that AS of the *EHMT2* gene may be related to spermatogenesis in Mongolian horses. However, the fundamental mechanism of as in spermatogenesis remains to be investigated. In this study, we explored the regulatory effect of AS events in the *EHMT2* gene on spermatogenesis in Mongolian horses. These findings lay a theoretical foundation for the study of the role of AS in spermatogenesis and provide new ideas for genetic breeding improvements.

## 2. Materials and Methods

### 2.1. Testis Collection and Cell Culture

All animal work was performed and approved by the Institutional Animal Care and Use Committee of Inner Mongolia Agricultural University (NO.NND2022047). Testicular tissue samples were obtained from Mongolian horses sourced from the Equus Research Center at Inner Mongolia Agricultural University. The testicular Sertoli cells of Mongolian horses were provided by a laboratory [22,23,24].

### 2.2. Immunofluorescence

Sertoli cells were fixed with 4% paraformaldehyde (Solarbio, Beijing, China) for 30 min at room temperature and then permeabilized in 0.5% Triton X-100 (Sigma, St. Louis, MO, USA) for 30 min. After blocking at 37 °C for 1 h with 5% bovine serum albumin (Sigma, St. Louis, MO, USA), the Sertoli cells were incubated with *G9a*/*EHMT2* Rabbit mAb (C6H3, Abcam, Cambridge, UK) at 4 °C overnight. Then, the corresponding secondary antibody, Rabbit lgG (H+L) Secondary Antibody Donkey anti-Mouse Alexa Fluor^TM^ 488 (R37118, Thermo Fisher Scientific, Waltham, MA, USA), was added. The mixture was incubated at 37 °C for 1 h. The cell nuclei were stained with 4,6-diamidino-2-phenylindole (DAPI) (Beyotime, Shanghai, China) for 30 min.

### 2.3. Analysis of the Predicted Protein Sequence

The ExPASy translation module (https://web.expasy.org/translate/, accessed on 29 May 2024) was used to translate the non-AS and AS nucleotide sequences of *EHMT2* to amino acid sequences. Protein secondary structures were predicted using SOPMA (https://npsa-prabi.ibcp.fr/cgi-bin/npsa_automat.pl?page=/NPSA/npsa_sopma.html, accessed on 31 May 2024), and three-dimensional model prediction was conducted via the Swiss-Meier program on the basis of known homologous structures.

### 2.4. Construction of the Lentiviral Vector

The Mongolian horse *EHMT2* gene sequence was obtained by sequencing. The lentiviral overexpression vectors with AS events and no AS of the *EHMT2* gene, named EX24 and EX24-1, respectively, were synthesized by GENEWIZ, Inc., Suzhou, China.

### 2.5. Cell Transfection

Sertoli cells were cultured in a culture dish, and transfection was initiated when the cells reached 50–60% confluence. An appropriate amount of lentivirus and culture medium was added. The culture dish was placed in a CO_2_ incubator at 37 °C. After 24 h, the transfection efficiency was measured.

### 2.6. Cell Proliferation Detected Using the CCK8 Assay

Sertoli cells were seeded in 96-well plates. Three replicates were established for each lentivirus at each time point in an incubator for 24 h (37 °C, 5% CO_2_). An appropriate amount of lentivirus and culture medium was added. Ninety microliters of fresh culture medium and CCK-8 solution (Meilun Bio, Dalian, China) were added at 0 h, 24 h, 48 h, and 72 h post infection. After 2 h, the absorbance at 450 nm was measured via a microplate reader (Thermo Scientific, Waltham, MA, USA) for three consecutive days. The data were analyzed and graphed using Excel software and GraphPad Prism 9 software.

### 2.7. RNA Isolation, cDNA Synthesis, and qRT–PCR Analysis

Total RNA was extracted from Sertoli cells by using TRIzol reagent (Takara, Kyoto, Japan) following the manufacturer’s procedure. cDNA synthesis was completed via the use of PrimeScriptTM RT Master Mix (Takara, Kyoto, Japan). Using Primer (5.0) software, primers were designed based on reference sequences from NCBI (https://www.ncbi.nlm.nih.gov, accessed on 3 June 2024) and subsequently synthesized by Sangon Biotech iotech Co., Ltd. (Shanghai, China). Glyceraldehyde-3-phosphate dehydrogenase (GAPDH) was selected as the internal reference gene for RT–PCR, RT–PCR was performed with TB Green TM Premix Ex TaqTM II (Tli RNaseH Plus) (Takara, Kyoto, Japan). Differences in gene expression levels between groups were analyzed using the 2^−ΔΔCt^ method. All experiments were performed with a minimum of three independent replicates, with the data presented as the means ± standard deviations. Intergroup differences were statistically evaluated using GraphPad Prism 9.0 software, with statistical significance set at *p* < 0.05. The sequences of primers used are listed in Supplementary Table S1.

## 3. Results

### 3.1. EHMT2 Expression in Sertoli Cells

**Figure 1:**
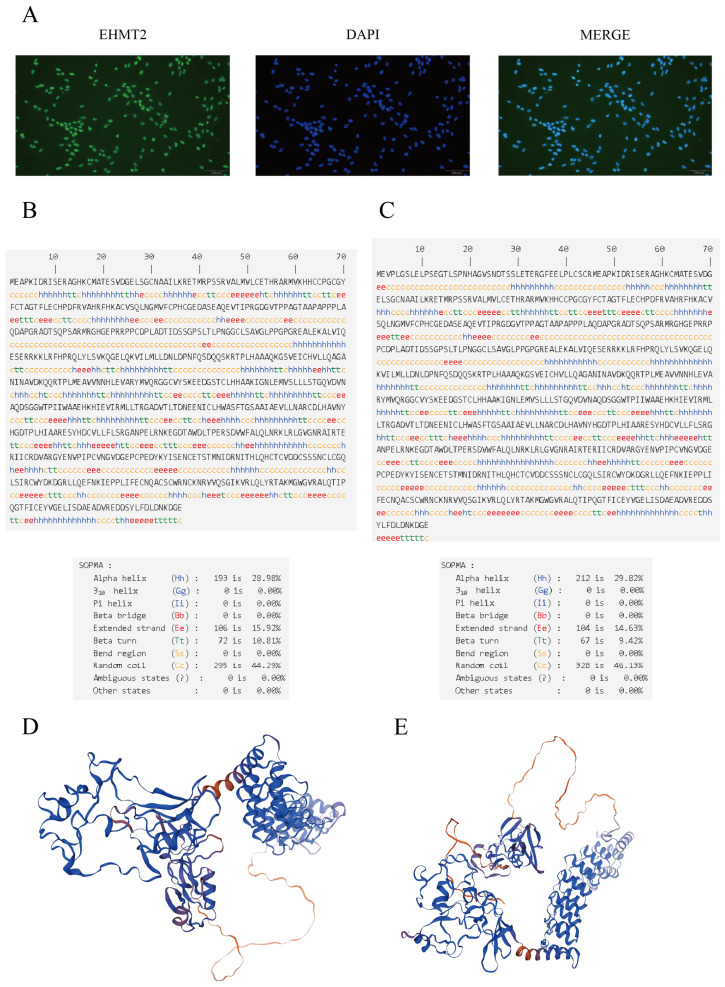
Subcellular localization of EHMT2 and high-resolution structural predictions of alternative splicing events and no alternative splicing. The localization of EHMT2 in Sertoli cells was determined by immunofluorescence (**A**). Secondary structure of the protein encoded by the *EHMT2* gene with and without AS ((**B**) and (**C**), respectively). Three-dimensional models for the proteins obtained with and without AS of the *EHMT2* gene ((**D**) and (**E**), respectively).

To precisely detect the localization and expression of *EHMT2* in Sertoli cells, we examined *EHMT2* expression in the testis by immunostaining with a *G9A/EHMT2* rabbit mAb (C6H3). *EHMT2* was found to be expressed in the nucleus of Sertoli cells (Figure 1A).

### 3.2. Predicted Secondary Structure of the EHMT2 Protein

The predicted secondary structure of the protein encoded by the *EHMT2* gene with AS included 28.98% alpha helix (Hh), 10.81% beta turn (Tt), 15.92% extended strand (Ee), and 44.29% random coil (Cc) structures, and that without AS included 29.82% alpha helix (Hh), 9.42% beta turn (Tt), 14.63% extended strand (Ee), and 46.13% random coil (Cc) structures (Figure 1B,C). Three-dimensional models of the proteins encoded by the *EHMT2* gene with and without AS were constructed (Figure 1D,E).

### 3.3. Lentiviral Vector Construction

**Figure 2:**
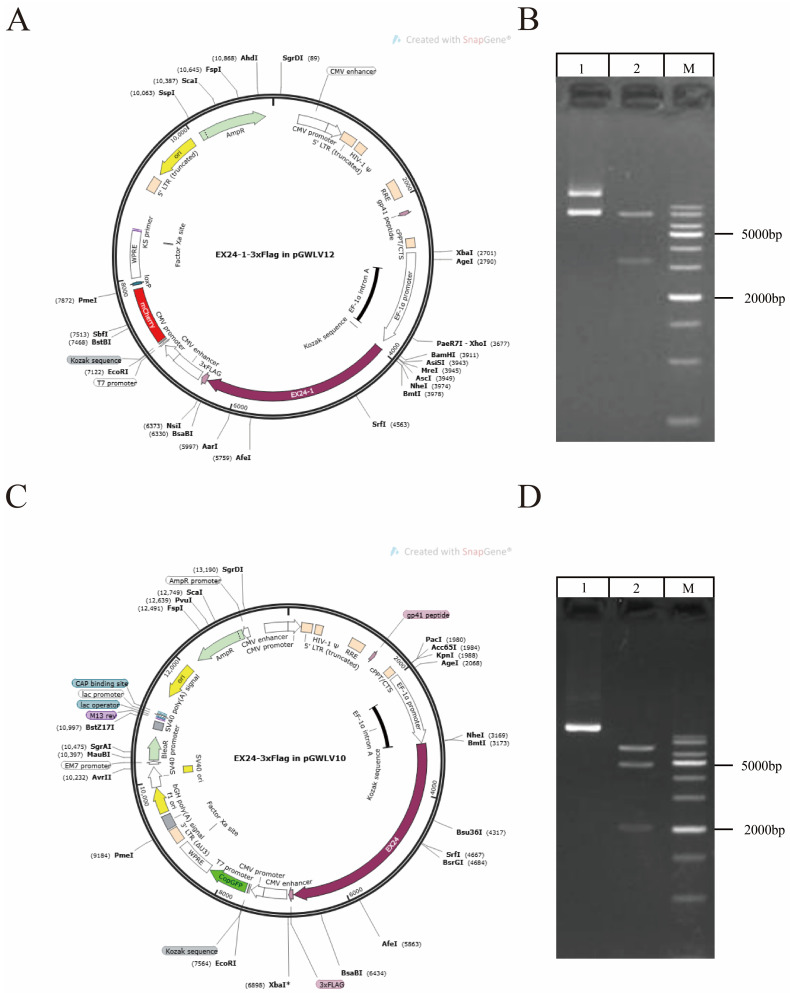
Construction of the lentiviral vector. Map of the construction of the overexpression lentiviral vector EX24-1. (**A**) Map of the constructed EX24-overexpressing lentiviral vector (**C**). Electrophoretic map of the EX24-1-overexpressing lentiviral vector (**B**). Electrophoretic map of the EX24-overexpressing lentiviral vector (**D**).

Maps of the two lentiviral vectors for overexpression are presented (Figure 2A,C). The EX24 and EX24-1 vectors encoded red and green fluorescent proteins and were 8790 bp and 8685 bp in length, respectively. The lengths of the predicted gene fragments and target bands obtained were consistent, which confirmed that the vectors were successfully constructed (Figure 2B,D).

### 3.4. Effects of the Two Lentiviruses on SERTOLI Cells

**Figure 3:**
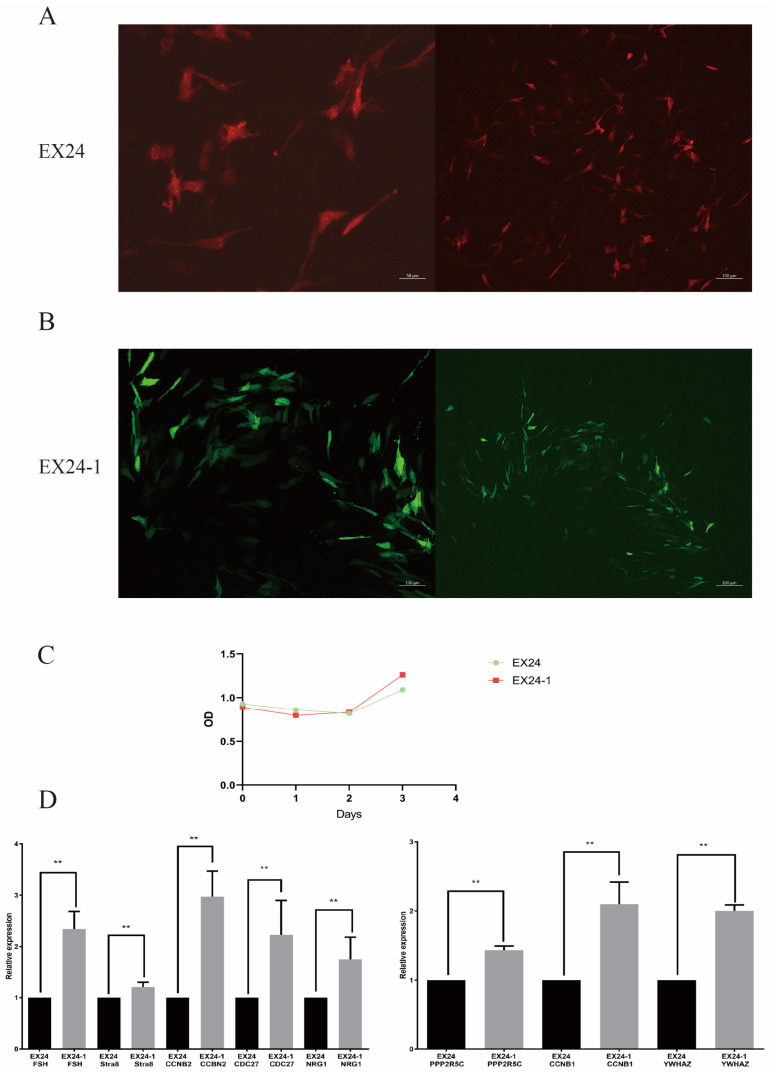
Effects of the two lentiviruses on Sertoli cells. Fluorogram after lentiviral transduction of EX24-1 cells (**A**). Fluorogram after lentiviral transduction of EX24 cells (**B**). Cell proliferation of the two types of cells (**C**). RT–PCR was used to detect the expression of the spermatogenesis-related genes *FSH*, *Stra8*, *CCNB2*, *CDC27*, *NRG1*, *PPP2R5C*, *CCNB2*, and *YWHAZ* in the two types of cells (**D**). ** indicates *p* < 0.01.

The Sertoli cells were infected with two lentiviral vectors at optimal doses, resulting in significant expression while maintaining the cells in their optimal state. Transfection was conducted for 24, 48, and 72 h, with the best transfection state observed at 72 h (Figure 3A,B). Photographs were taken to record the results. The infection efficiency was approximately 70%.

### 3.5. Cell Proliferation Detected of Sertoli Cells by the CCK-8 Assay

The effect of AS of the *EHMT2* gene on the proliferation of Sertoli cells was determined via the CCK8 method. The OD values measured within 3 days were plotted as a growth curve (Figure 2C), with EX24-1 exhibiting peaks on day 3.

### 3.6. qRT–PCR

The expression levels of spermatogenesis-related genes were measured in the two groups. The results revealed that the expression levels of the *FSH*, *Stra8*, *CCNB2*, *CDC27*, *NRG1*, *PPP2R5C*, *CCNB2*, and *YWHAZ* genes in the AS group were greater than those in the control group (Figure 3D). These results indicate that AS events in *EHMT2* affect gene expression and thus affect spermatogenesis.

## 4. Discussion

Epigenetic regulation of gene expression by covalent modification of histones is important for germline cell development. In mammals, histone H3 lysine 9 (*H3K9*)-specific histone methyltransferases (HMTases) play a critical role [19]. In addition, two kinds of proteins are encoded by *EHMT2* genes in mammals: *EHMT1* encodes *GLP*, and *EHMT2* encodes *G9a* [25]. *G9a* plays critical roles in germline cell development in mammals. For example, it has been reported *G9a* knockout is embryonically lethal in mice [26]. Both male and female germline cells of *G9a* mutant mice cannot undergo meiosis [20], indicating an essential role of *G9a* during germ cell development. We found that *EHMT2* was expressed in Sertoli cell nuclei, as shown by immunofluorescence. Therefore, Sertoli cells can be used to verify the function of the *EHMT2* gene.

Sertoli cells, which envelop spermatogenic cells at distinct developmental stages, serve as the sole somatic cells maintaining direct contact with germ cells throughout their maturation. Sertoli cells regulate spermatogenesis by secreting proliferation- and differentiation-associated trophic factors to provide physiological and metabolic support for germ cells, while simultaneously establishing the blood–testis barrier (BTB) [27,28]. Studies demonstrate that Sertoli cells regulate androgen-binding protein (Androgen-Binding Protein, ABP) metabolism via highly active autophagic activity, thereby establishing a high-concentration androgen milieu essential for germ cell development and critically influencing spermatogenesis [29]. The number of Sertoli cells in the adult testis determines the population of germ cells supportable during spermatogenesis, modulates the progression of spermatogenesis, and ultimately governs daily sperm output [30,31].

According to the Human Genome Project (HGP), which released a draft of the human genome in 2001, the entire human genome is approximately 2.91 G bp, equivalent to more than 35,000 genes [32,33,34]. This is not what scientists had predicted [35,36]. The diversity of gene transcription plays a very important role in this phenomenon. The main mechanisms for increasing the diversity and quantity of gene transcripts and proteins include DNA recombination, RNA editing, and AS, with AS serving as the primary mechanism. AS is commonly observed in the testes of mammals and affects spermatogenesis by influencing genes related to sperm production. For example, the RNA binding protein 9 gene (*RANBP9*) regulates AS events in spermatogenic cells and is closely related to spermatogenesis [37]. Transcriptomic analysis of mouse spermatogenesis has revealed over 13,000 AS events, indicating that AS is a key driver of cell differentiation events during spermatogenesis [38].

In this study, we explored the regulatory effect of AS of the *EHMT2* gene on spermatogenesis in Mongolian horses. These findings lay a theoretical foundation for the study of AS in spermatogenesis and provide new ideas for genetic breeding improvements. Many studies have shown that AS plays an important role in regulating the expression and function of related genes in germ cells. Spermatogenesis plays a very important role in the life process of male animals and is a prerequisite for the stable transmission of genetic information from parents to the next generation. The secondary structures of the proteins encoded by the *EHMT2* gene with and without AS exhibit marked structural divergences, suggesting potential variations in the encoded protein isoforms. Further investigation is warranted to elucidate the functional implications of these structural differences.

In this study, we selected key genes involved in spermatogenesis and examined their differential expression in two types of cells. The results reveal that the expression levels of the target genes were significantly greater in the cells with AS. These findings indicate that AS of the *PPP2R5C* gene indeed upregulates the expression of meiosis-related genes, thereby affecting spermatogenesis. *PPP2R5C* is a subunit of protein phosphatase 2A (PP2A), and mutation if this gene can affect cell proliferation and accelerate apoptosis. Downregulation of *PPP2R5C* can cause cell cycle arrest, thereby promoting apoptosis [39]. The *CCNB1* and *CCNB2* genes are expressed at various stages of spermatogonial mitosis. When *CCNB1* and *CCNB2* are knocked out in the testes of experimental male mice, spermatogonia cannot proliferate normally, and apoptosis increases, resulting in male mouse infertility [40,41]. Male FSH-R knockout (FORKO) mice present fewer Sertoli cells and many that are structurally abnormal, and, as a consequence, fewer germ cells. Lower levels of serum testosterone (T) and androgen binding protein (ABP) also occur in these mice, along with reduced fertility. Successful spermatogenesis occurs through precise regulation of gene expression. Stimulated by retinoic acid gene 8 (*Stra8)* is a key molecule involved in meiosis initiation and plays an important role in spermatogenesis [42]. The major function of Stra8 in spermatogonial differentiation is also involved in meiotic initiation, the establishment and maintenance of SSCs, cell proliferation, self-renewal, undifferentiation, and other physiological processes [43]. The *YWHA*-binding proteins in sperm can be classified as those involved in fertilization, acrosome reactions, energy metabolism, protein folding, and ubiquitin-mediated proteolysis. A subset of these putative *YWHA*-binding proteins contains known amino acid consensus motifs, not only for *YWHA* binding, but also for *PPP1C* binding [44].

## 5. Conclusions

To study the regulatory role of AS events in the *EHMT2* gene in spermatogenesis in Mongolian horses, this study first examined the localization of the *EHMT2* gene in testicular support cells and then predicted the higher-order structures of sequences with and without AS. Two types of overexpressed lentiviral vectors were constructed for the *EHMT2* gene, one with AS and one without AS, to infect support cells. The proliferation and activity of the infected cells were detected using CCK8, and the differential expression of spermatogenesis-related genes in the two types of support cells was analyzed via qRT–PCR. This study fills the knowledge gap regarding alternative splicing mechanisms in Mongolian horse spermatogenesis. The findings of this study could provide a theoretical foundation for advancing Mongolian horse breeding programs, thereby enabling a more comprehensive and in-depth investigation into the regulatory mechanisms of alternative splicing events in spermatogenesis.

## Data Availability

The original contributions presented in this study are included in the article/Supplementary Materials. Further inquiries can be directed to the corresponding authors.

## Supplementary Materials

The following supporting information can be downloaded at: https://www.mdpi.com/article/10.3390/ani15081106/s1, Table S1. RT-qPCR primers.
